# Antimicrobial prescription practices for outpatients with uncomplicated cystitis in Japan

**DOI:** 10.1038/s41598-022-09946-8

**Published:** 2022-04-08

**Authors:** Misa Takahashi, Hideharu Hagiya, Tsukasa Higashionna, Yasuhiro Nakano, Kota Sato, Yuto Haruki, Mai Haruki, Hiroyuki Honda, Hiroko Ogawa, Keigo Ueda, Fumio Otsuka

**Affiliations:** 1 Department of General Medicine, Okayama University Graduate School of Medicine, Dentistry and Pharmaceutical Sciences, 2-5-1 Shikata-cho, Kita-Ku, Okayama, 700-8558 Japan; 2 Department of Pharmacy, Okayama University Hospital, Okayama, 700-8558 Japan; 3 Department of Neurology, Brain Attack Center Ota Memorial Hospital, Fukuyama, 720-0825 Japan; 4 Department of Pharmacy, Tsuyama Chuo Hospital, Okayama, 708-0841 Japan

**Keywords:** Diseases, Health care, Medical research, Risk factors, Signs and symptoms, Urology

## Abstract

To promote antimicrobial stewardship, we studied antimicrobial prescription rates for uncomplicated cystitis, a common outpatient disease requiring antibiotic treatment. This multicenter retrospective study was performed from January 1, 2018, to December 31, 2020, in Japan, targeting outpatients aged ≥ 20 years whose medical records revealed International Classification of Diseases (ICD-10) codes suggesting uncomplicated cystitis (N300). The data of 1445 patients were collected and that of 902 patients were analyzed. The overall median patient age was 71 years and a proportion of those aged less than 50 years was 18.8% with a female dominance (82.6%). Antimicrobials were prescribed for 884 patients (98.0%) and a total of 623 patients (69.1%) were treated with broad-spectrum drugs, including fluoroquinolones (36.0%), third-generation cephalosporins (29.9%) and faropenem (3.1%). A logistic regression model revealed that the broad-spectrum agents were significantly prescribed for the older patients, male patients, and those who visited internists. Recurrence was observed in 37 (4.1%) cases, and the multivariate analysis suggested any of age, sex, or antimicrobial types were not associated with the recurrence. Collectively, approximately two-thirds of antimicrobials prescribed for uncomplicated cystitis were broad-spectrum agents. The present data would be an indicator for antimicrobial prescriptions in uncomplicated cystitis in Japan.

## Introduction

With an unprecedented spread of antimicrobial resistance (AMR) pathogens worldwide, the global AMR Action Plan was issued in 2015, emphasizing the importance of appropriate use of antimicrobial agents in medical practices^1^. For international comparison, the average daily use of antimicrobial agents per thousand population is considered as an indicator of appropriate use of antimicrobial agents. In 2013, the antimicrobial use in Japan was approximately 15.8 per thousand population per day^2^, which was the second-lowest rate after that of Germany when compared to the developed countries of the European Union^2,3^. However, the prescribings of oral broad-spectrum antimicrobials, including fluoroquinolones, cephalosporins, and macrolides, was still higher than those in other countries^2^. To combat the domestic spread of AMR in Japan, the Japanese national AMR Action Plan was launched in 2016 to further reduce the use of antimicrobial agents by 33% by 2020 compared to that prescribed in 2013^2^. However, it is still unclear whether the usage of antimicrobial agents has been successfully optimized in Japan.

Urinary tract infections (UTIs), especially cystitis, are representative of common infectious diseases that clinicians frequently encounter in daily practice. In general, 50–70% of females suffer from cystitis at some point in life^4,5^, and over 250 million people are globally involved with the disease every year^6^. Recurrence is also common, and 30–44% of females report a repeated episode of cystitis within three months^7^. Thus, antimicrobials are frequently prescribed for the treatment of cystitis in outpatient settings. Additionally, clinical isolations of AMR pathogens, such as extended-spectrum beta-lactamase producing, fluoroquinolone-resistant, or carbapenem-resistant gram-negative bacilli, have been increasingly reported in patients with UTIs^8,9^. These findings imply the need for antimicrobial stewardship (AMS) to be further evaluated and promoted in this field.

European and American guidelines have recommended sulfamethoxazole-trimethoprim combination, fosfomycin, nitrofurantoin, and pivmecillinam for the treatment of uncomplicated cystitis^8–10^. Nitrofurantoin and pivmecillinam are currently unavailable in Japan, and the JAID/JSC Infectious Disease Treatment Guide recommends fluoroquinolones as first-line drugs for uncomplicated cystitis instead^11^. To avoid overprescription, the use of fluoroquinolones should be limited to selected cases, such as complicated or severe UTIs. However, the fluoroquinolones are frequently prescribed to patients with umcomplicated cystitis presumably because of its superiority in both pharmacokinetic and pharmacodynamics properties^12^. Consequently, isolation rates of fluoroquinolone-resistant *Escherichia coli* have increased from 24% in 2007 to 41.5% in 2020 in Japan according to the national surveillance data^13,14^. Similarly, administration of third-generation cephalosporins is recommended in the Japanese guideline^11^, which also potentially induces the development of AMR pathogens.

A recent, national surveillance study based on domestic administrative data demonstrated that fluoroquinolones and third-generation cephalosporins accounted for more than 90% of the antimicrobials prescribed for female patients aged ≥ 15 years with uncomplicated cystitis^12^. This was highly indicative of the need for proceeding with AMS in outpatient UTIs treatment in Japan; however, due to the nature of the database, the study lacked clinical data endorsing a definitive diagnosis of UTIs. This study aimed to investigate antimicrobial prescriptions for uncomplicated cystitis and underlying factors for such prescriptions based on the retrospective analysis of medical records.

## Results

**Figure 1:**
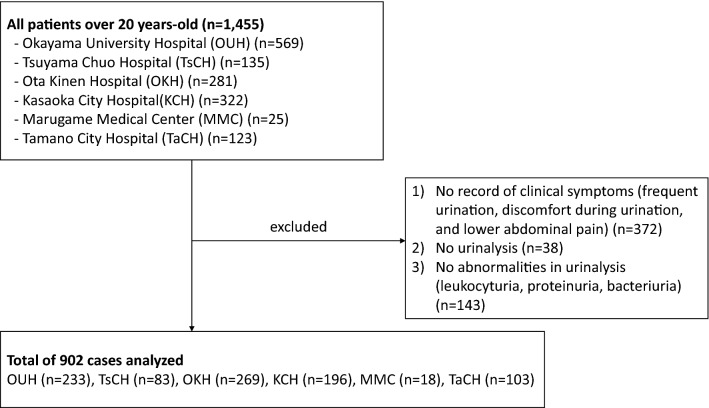
Overall study flow.

We collected the data of 1455 patients from six medical institutions. Of these, 902 cases, for which the clinical symptoms and results of urinalysis were well documented in the medical records, were included for the analysis. The numbers of patients whose data were collected from each institute are listed as follows: Okayama University Hospital (n = 233), Tsuyama Chuo Hospital (n = 83), Brain Attack Center Ota Memorial Hospital (n = 269), Kasaoka City Hospital (n = 196), Marugame Medical Center (n = 18), and Tamano City Hospital (n = 103) (Fig. 1). The overall median patient age [IQR] was 71 [57, 79] years and a proportion of those aged less than 50 years was 18.8% (Table 1) with a female dominance (745 females [82.6%] and 157 males [17.4%]). The numbers (percentages) of patients concerning consulting departments were as follows: 85 cases (9.4%) in internal medicine, 670 cases (74.3%) in urology, and 44 cases (4.9%) in other departments (Gynecology, 30; Surgery, 13; Dental, 1). The consulting departments for 103 cases (11.4%) were unknown in the medical records. Of the 902 eligible cases, urine culture was submitted in 616 (68.3%) of the cases before the antimicrobial prescription, of which 573 patients (93.0%) tested positive and *E. coli* (58.4%) was the most common organism detected (Table 2).

**Table 1:** The numbers and percentages of background data of the eligible cases in each medical institute.

	Overall	Medical institutes					
		OUH	TsCH	OMH	KCH	MMC	TaCH
The number of cases	902	233	83	269	196	18	103
Median age [IQR], years	71 [57, 79]	71 [60, 76]	68 [46, 81]	67 [48, 79]	76 [67, 82]	47 [29, 74]	71 [66, 82]
**Age group (%)**							
< 50 years	170 (18.8)	34 (14.6)	25 (30.1)	73 (27.1)	20 (10.2)	10 (55.6)	6 (7.1)
≥ 50 years	732 (81.2)	199 (85.4)	58 (69.9)	196 (72.9)	176 (89.8)	8 (44.4)	79 (92.9)
Sex (F/M) (%)	745/157 (82.6/17.4)	142/91 (60.9/39.1)	70/13 (84.3/15.7)	246/23 (91.4/8.6)	171/25 (87.2/12.8)	17/1 (94.4/5.6)	99/4 (96.1/3.9)
**Consulting department (%)**							
Internal medicine	85 (9.4)	31 (13.3)	6 (7.2)	2 (0.7)	38 (19.4)	8 (44.4)	0
Urology	670 (74.3)	175 (75.1)	72 (86.8)	266 (98.9)	150 (76.5)	7 (38.9)	0
Others	44 (4.9)	27 (11.6)	5 (6.0)	1 (0.4)	8 (4.1)	3 (16.7)	0
Unrecorded	103 (11.4)	0	0	0	0	0	103 (100)
**Bacterial culture (%)**							
Tested	616 (68.3)	121 (51.9)	69 (83.1)	239 (88.9)	107 (54.6)	2 (11.1)	78 (75.7)
Not tested	286 (31.7)	112 (48.1)	14 (16.9)	30 (11.2)	89 (45.4)	16 (88.9)	25 (24.3)

The International Classification of Diseases (ICD-10) codes were endorsed in May 1990 by the Forty-third World Health Assembly to develop the diagnostic classification standard for all clinical and research purposes. *IQR* interquartile range, *ND* no data.

**Table 2:** Breakdown list of the isolated pathogens.

Pathogens	Overall (%)	Age group		Sex	
		≤ 50 years (%)	> 50 years (%)	Female (%)	Male (%)
**Gram-negative rods**					
*E.coli* (ESBL 39, non-ESBL 306)	345 (58.4)	64 (55.7)	281 (58.9)	317 (62.6)	28 (32.9)
*Klebsiella pneumoniae* (ESBL 1, non-ESBL 19)	20 (3.4)	1 (0.9)	19 (4.0)	16 (3.2)	4 (4.7)
*Proteus* species	16 (2.7)	1 (0.9)	15 (3.1)	13 (2.6)	3 (3.5)
*Citrobacter* species	10 (1.7)	1 (0.9)	9 (1.9)	8 (1.6)	2 (2.4)
*Pseudomonas aeruginosa*	10 (1.7)	0 (0.0)	10 (2.1)	4 (0.8)	6 (7.1)
*Klebsiella oxytoca*	7 (1.2)	0 (0/0)	7 (1.5)	5 (1.0)	2 (2.4)
*Enterobacter* species	5 (0.8)	1 (0.9)	4 (0.8)	2 (0.4)	3 (3.5)
**Gram-positive cocci**					
Coagulase-negative Staphylococci	30 (5.1)	9 (7.8)	21 (4.4)	25 (4.9)	5 (5.9)
*Enterococcus* species	21 (3.6)	2 (1.7)	19 (4.0)	9 (1.8)	12 (14.1)
*Streptococcus* species	38 (6.4)	11 (9.6)	27 (5.7)	32 (6.3)	6 (7.1)
*Staphylococcus aureus* (MSSA 8, MRSA 3)	11 (1.9)	2 (2.6)	9 (1.9)	10 (2.0)	1 (1.2)
Others	78 (13.2)	22 (19.1)	56 (11.7)	65 (12.8)	13 (15.3)

*ESBL* extended-spectrum beta-lactamases, *MSSA* methicillin-sensitive *S. aureus*, *MRSA* methicillin-resistant *S. aureus.*

**Figure 2:**
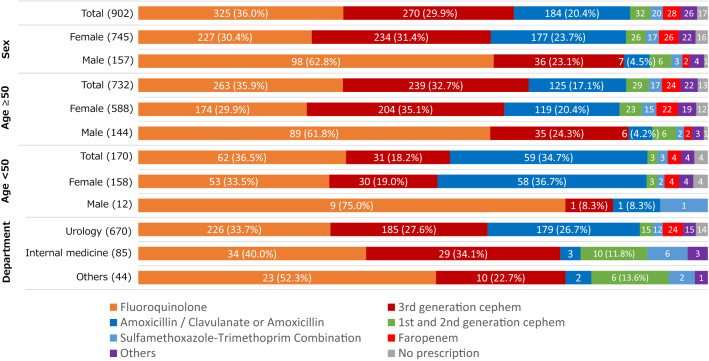
These are details of antimicrobial prescriptions for cystitis by sex, age group, and consulting department.

Frequency and details of antimicrobial prescriptions concerning sex, age group, and consulting department were summarized in Table 3 and Fig. 2. Overall, antimicrobials were prescribed for 884 patients (98.0%). No differences were observed in the proportions of antimicrobial prescriptions concerning age, sex, and the consulting department. Fluoroquinolones were the most commonly prescribed agents (n = 325, 36.0%), followed by third-generation cephalosporins (n = 270, 29.9%), and a total of 623 patients (69.1%) were treated with the broad-spectrum drugs. Amoxicillin (AMPC) with or without clavulanate (CVA) (n = 184, 20.4%), first- or second-generation cephalosporins (n = 32, 3.5%), and sulfamethoxazole-trimethoprim (n = 20, 2.2%) were less frequently administered; a total of 26.2% of the patients were treated with these narrow-spectrum antimicrobials. When stratified by age, the numbers of patients in the younger and older age groups were 170 (18.8%) and 732 (81.2%), respectively. Fluoroquinolones were most frequently prescribed in both the younger group (36.5%) and the older group (35.9%). The second most commonly prescribed agents in the younger group were AMPC with or without CVA (34.7%), followed by third-generation cephalosporins (18.2%). While in the older age group, third-generation cephalosporins (32.7%) were prescribed more often than AMPC with or without CVA (17.1%). When stratified by sex, more than half (62.8%) of the male cases were prescribed fluoroquinolones, followed by third-generation cephalosporins (23.1%). Hence, 86.6% of the male patients were subjected to broad-spectrum antimicrobial treatment. In contrast, fluoroquinolones (30.4%), third-generation cephalosporins (31.4%), and AMPC with or without CVA (23.7%) were almost equally prescribed to female patients. When stratified by the consulting department, fluoroquinolones (40.0%) and third-generation cephalosporins (34.1%), but not AMPC with or without CVA, were the major drugs prescribed to patients in the internal medicine department. The first and second-generation cephalosporins (11.8%) and sulfamethoxazole-trimethoprim combination (7.1%) were rather frequently used. In urology, patients were most frequently prescribed fluoroquinolones (33.7%), followed by third-generation cephalosporins (27.6%) and AMPC with or without CVA (26.7%). Faropenem was prescribed to 24 patients (3.6%) by urologists, which was not prescribed in the internal medicine department.

**Table 3:** Numbers and proportions of antimicrobial prescriptions for cystitis by age group, sex, and consulting department.

	Visits	Antimicrobial prescription			
	Numbers	Numbers	% (95% CI)	Odds ratio (95% CI)	*p* values
Overall	902	884	98.0% (96.9–98.8)	–	–
**Age group (%)**					
< 50 years	170 (18.8)	166	97.6% (94.1–99.4)	Reference	–
≥ 50 years	732 (81.2)	718	98.1% (96.8–99.0)	1.24 (0.29–4.00)	0.76
**Sex (%)**					
Female	745 (82.6)	728	97.7% (96.4–98.7)	Reference	–
Male	157 (17.4)	156	99.4% (96.5–100)	3.64 (0.56–153.1)	0.34
**Consulting department**					
Internal medicine	85 (9.4)	84	98.8% (93.6–100)	Reference	–
Urology	670 (74.3)	656	97.9% (96.5–98.9)	0.56 (0.01–3.76)	1.00
Others	44 (4.9)	44	100.0% (92.0–100)	Not compared	–
Unrecorded	103 (11.4)	–	–	–	–

*CI* confidence interval.

The results of univariate and multivariate analysis for broad-spectrum antimicrobial prescriptions for uncomplicated cystitis are summarized in Table 4. Compared to the younger patients, the older patients received broad-spectrum drugs (57.1% vs. 71.9%; OR [95% CI], 1.83 [1.23–2.71]). Compared to female patients, male patients were treated with broad-spectrum antimicrobial drugs (65.4% vs. 86.6%; OR [95% CI], 4.68 [2.66–8.25]). With respect to the consulting department, compared to the internal medicine department, broad-spectrum antimicrobials were less frequently prescribed in the urology department (74.1% vs. 64.9%; OR [95% CI], 0.53 [0.31–0.92]).

**Table 4:** Univariate and multivariate analysis for broad-spectrum antimicrobial prescriptions for uncomplicated cystitis.

	Visits	Broad-spectrum	Narrow-spectrum		Univariate analysis		Multivariate analysis	
	Number (%)	Number	% (95% CI)	Number (%)	OR (95% CI)	*p* values	OR (95% CI)	*p* values
Overall	902	623	69.1% (65.9–72.1)	236 (26.2%)	–	–	–	–
**Age group**								
< 50 years	170 (18.8)	97	57.1% (49. 64.6)	65 (38.2%)	Reference	–	Reference	–
≥ 50 years	732 (81.2)	526	71.9% (68.4–75.1)	171 (23.4%)	1.92 (1.34–2.75)	< 0.001	1.83 (1.23–2.71)	0.003
**Sex**								
Female	745 (82.6)	487	65.4% (61.8–68.8)	220 (29.5%)	Reference	–	Reference	–
Male	157 (17.4)	136	86.6% (80.3–91.5)	16 (10.2%)	3.43 (2.09–5.86)	< 0.001	4.68 (2.66–8.25)	< 0.001
**Department**								
Internal medicine	85 (9.4)	63	74.1% (63.5–83.0)	19 (22.4%)	Reference	–	Reference	–
Urology	670 (74.3)	435	64.9% (61.2–68.5)	206 (30.7%)	0.65 (0.37–1.10)	0.11	0.53 (0.31–0.92)	0.025
Others	44 (4.9)	33	75.0% (59.7–86.8)	10 (22.7%)	Not compared	–	Not compared	–

*CI* confidence interval, *OR* odds ratio. Total number of 902 cases were subjected to the univariate analysis (Chi-square test) and multivariate analysis (logistic regression model).

We finally investigated potential factors responsible for the recurrence of cystitis (Table 5). The total number of patients demonstrating recurrence was 37 (4.1%). The recurrence rates in the younger and the older group were 2.4% and 4.5%, respectively, and those in the female and male patients were 4.4% and 2.5%, respectively. Narrow-spectrum antimicrobials were prescribed in 3.0% of recurrence cases, while broad-spectrum antimicrobials were prescribed in 4.2% of the cases. Univariate analysis did not suggest any statistical differences among each category. We incorporated age, sex, and antimicrobial types into the logistic regression model, and found that these factors were not statistically related to the recurrence. Particularly, compared to the narrow-spectrum antimicrobials, the broad-spectrum agents were not associated with recurrence (3.0% vs. 4.2%; OR [95% CI], 1.42 [0.60–3.35]).

**Table 5:** Univariate and multivariate analysis for recurrence of cystitis.

	Visits	Recurrence		No recurrence	Univariate analysis		Multivariate analysis	
	Number	Number	% (95% CI)	Number (%)	OR (95% CI)	*p* values	OR (95% CI)	*p* values
Overall	900	37	4.1% (2.9–5.6)	863 (95.9%)	–	–	–	–
**Age group**								
< 50 years (%)	169 (18.8)	4	2.4% (0.6–5.9)	165 (97.6%)	Reference	–	Reference	–
≥ 50 years (%)	731 (81.2)	33	4.5% (3.1–6.3)	698 (95.5%)	1.95 (0.68 –7.68)	0.28	2.42 (0.72–8.09)	0.15
**Sex**								
Female (%)	743 (82.6)	33	4.4% (3.1–6.2)	710 (95.6%)	Reference	–	Reference	–
Male (%)	157 (17.4)	4	2.5% (0.7–6.4)	153 (97.4%)	0.56 (0.14–1.62)	0.38	0.54 (0.19–1.59)	0.27
**Antimicrobials**								
Narrow-spectrum	236 (26.2)	7	3.0% (1.2–6.0)	229 (97.0%)	Reference	–	Reference	–
Broad-spectrum	623 (69.2)	26	4.2% (2.7–6.1)	597 (95.8%)	1.42 (0.59–3.94)	0.55	1.42 (0.60–3.35)	0.42

*OR* odds ratio.

Of the total 902 cases, 2 cases were excluded from the analysis since they were lack of information on the recurrence. Consequently, 900 cases were subjected to the univariate analysis (Chi-square test) and multivariate analysis (logistic regression model).

## Discussion

In this multi-centered study, we examined the antimicrobial prescriptions for patients diagnosed with uncomplicated cystitis. In comparison with the administrative database study reporting that more than 90% of the antimicrobials prescribed for uncomplicated cystitis were either fluoroquinolones or third-generation cephalosporins^12^, the proportion of broad-spectrum antimicrobials in our cohort was lower at neraly two-thirds of the cases. The broad-spectrum agents were prescribed frequently in the older group (≥ 50 years), male patients, and by internists. Of note, neither age, sex, nor antimicrobial types were associated with the recurrence of uncomplicated cystitis.

The primary aim of this study was to elucidate the prescribing rates of the antimicrobials for uncomplicated cystitis by directly drawing clinical data from medical records. Based on the health insurance claims data^12^, fluoroquinolones (52.7%) and third-generation cephalosporins (36.9%) accounted for most of the prescriptions for female patients aged ≥ 15 years with uncomplicated cystitis. This result indicates that the prescription rates of broad-spectrum antimicrobial agents for cystitis in female patients reach nearly 90% in Japan. However, our clinical database demonstrated that the overall prescription rates of fluoroquinolones and third-generation cephalosporin were comparatively lower at 36.0% and 29.9%, respectively. Focusing on female patients, those were prescribed to 30.4% and 31.4% of the cases, respectively. Including faropenem, the prescription rate of broad-spectrum antimicrobials for uncomplicated cystitis in females was 65.4%, which is still high but much lower than the data described in the administrative database study^12^. A potential explanation for the differences in the prescription rates of broad-spectrum antimicrobials includes a discrepancy in patient demographics. To collect data for patients with uncomplicated cystitis, however, we used ICD-10 code N300 similar to that employed in the previous study^12^. Thus, we believe that patients with similar clinical backgrounds were recruited. Upon examination of the patient age, more than 80% of the patients in our cohort were aged ≥ 50 years, while more than half of the patients in the administrative data were aged < 50 years. However, the high proportion of elderly patients could have resulted in more prescriptions of broad-spectrum antimicrobial agents, but this was not the case. While the previous study included teenagers (patients aged ≥ 15 years), our study involved only those aged 20 years and more. This difference, however, would rather not influence the manner of prescriptions remarkably, because fluoroquinolones are typically not recommended for pediatric patients by package inserts in Japan. Another possible factor for fewer prescriptions of the broad-antimicrobials in the present study may be an advancement in AMS at the participating medical institutes, although it was not fully measured.

Our study suggested that age factor potentially affects antimicrobial prescribing. In the previous study^12^, the broad-spectrum antimicrobial prescription rates for uncomplicated cystitis were almost same; 91.1% in the younger group (< 50 years) and 90.1% in the older group (≥ 50 years). In contrast, the proportions of broad-spectrum antimicrobial prescriptions among the younger and older groups were 57.1% and 71.9% (OR, 95% CI 1.83 [1.23–2.71]) in our data, indicating that aged patients were more likely to be prescribed broad-spectrum drugs, which has also been observed in other studies^15^. This could be rationalized by the fact that aged people tend to have underlying diseases frequently, which are conceivably associated with development of complicated or severe UTIs.

Our data corroborated that the prescription of broad-spectrum drugs was significantly frequent in males than in females; 86.6% versus 65.4% (OR, 95% CI 4.68 [2.66–8.25]). Owing to the anatomical advantage, UTIs infrequently occur in males^16,17^. Male patients with UTIs generally have any of underlying urological abnormalities such as urinary tract stones/malignancy, neurogenic bladder, and ﻿benign prostatic hyperplasia^18–20^. Our observations may be attributed to this dissimilarity between the sexes in terms of vulnerability to UTIs. Considering the limitations of the feasibility of the study, we did not collect detailed data of patient characteristics, and hence, could not adjust their backgrounds.

A single-facility study suggested that organisms isolated from patients visiting urology department with uncomplicated cystitis tend to show resistance to various antibiotics compared to hospital-wide antibiograms^21^. Thus, we speculated that a higher number of broad-spectrum drugs would be prescribed by urologists in our study. However, our investigation found fewer prescriptions by the urologists, while more usages by internists. This can merely be inter-facility or inter-physician differences, which should be evaluated by future study.

Importantly, our multivariate analysis suggested that prescriptions of the broad-spectrum antimicrobials were not associated with the prevention of the recurrence of cystitis. Given AMS, broad-spectrum drugs, particularly fluoroquinolones, should not be prescribed for common diseases like uncomplicated cystitis. A recent meta-analysis based on the systematic review of 47 randomized controlled trials demonstrated the superiority of fluoroquinolones compared to that of other antimicrobial agents in terms of clinical remission rates, bacteriological eradication, emergence of drug resistance, and relapsing rates^22^. A retrospective population-based cohort study based on administrative health data extracted from six Canadian provinces also verified the advantages of fluoroquinolone prescriptions, such as fewer revisits of outpatients and emergency patients, hospital admission, and re-prescription of antimicrobials within 30 days^23^. However, fluoroquinolones have a variety of adverse drug effects, including QT elongation, glucose intolerance, retinal detachment, tendinitis, aortic aneurysm, and neurologic disorders^24^. Also, the increasing trend of clinical isolations of fluoroquinolone-resistant organisms in UTIs has been suggested by recent surveillance studies in Japan^13,14,21,25,26^. Although these facts would make us reluctant to treat patients with uncomplicated cystitis with fluoroquinolones, our data demonstrated that many such cases are still treated with the broad-spectrum drugs. Our analysis indicated that the administration of narrow-spectrum antimicrobials, including AMPC, first- or second-generation cephalosporins, and sulfamethoxazole-trimethoprim, is not associated with the recurrence, supporting the safety of these treatment for patients with uncomplicated cystitis.

Usages of antimicrobials for uncomplicated cystitis vary greatly from country to country. Accoding to national ambulatory datasets of the United States, almost half (49%) of female patients with uncomplicated cystitis was treated with fluoroquinolones, followed by sulfonamides (27%) and nitrofurantoin (19%)^27^. A population-based retrospective cohort study in England found that 73.8% of elderly patients with UTIs were prescribed either trimethoprim (54.7%) or nitrofurantoin (19.8%), while cephalosporins (11.5%), AMPC/CVA (9.5%), and fluoroquinolones (4.4%) were prescribed in fewer cases^28^. In a ﻿national registry-based study in Denmark, ﻿pivmecillinam (45.8%) was the most common antibiotic for acute lower UTIs, followed by sulfonamide (27.0%)^29^. These differences could partly be attributed to the discrepancy of recommendations in national guidelienes in each country. In fact, although the Japanese guideline suggested fluoroquinolones as the first choice^11^, the Infectious Diseases Society of America guideline recommended nitrofurantoin, trimethoprim-sulfamethoxazole, ﻿fosfomycin, and ﻿pivmecillinam ﻿for acute uncomplicated cystitis^8^. Moreover, national guidelines in 15 European countries have great variability in the selection of antibiotics^30^; *e.g.,* 10 different antimicrobials were recommended as the first-line therapy. This discrepancy in national guidelines may be due in part to the unavailability of drugs in each nation; in fact, nitrofurantoin and pivmecillinam are currently unavailable in Japan^31^. Difference of antibiogram in each region should also influence on the recommendation and selection of the drugs. ﻿To promote AMS and reduce the use of broad-spectrum antibiotics such as fluoroquinolones, review and reconsideration of the antimicrobials approved and distributed in each country is warranted.

The strength of the present study lies in the direct collection of clinical data from medical records. Previous larger studies were based on health insurance claims data^12^, and therefore, the validity of its clinical diagnosis was unreliable. However, there are several limitations to this study. First, despite the multi-centered database, the data of our cohorts were derived merely from six medical institutes. Thus, the generalizability of the study should be evaluated by larger investigations. Second, the ages of the patients were higher for cystitis in this study. This could be attributed to the fact that we primarily collected data from regional hospitals in rural areas where the population is aging rapidly. Third, information essential to the selection of antimicrobials, such as the history of medication allergies, was not collected. Fourth, the ICD-10 codes given in the medical records may be labeled just for convenience to not interrupt their antimicrobial orders. Finally, we did not investigate the duration of antimicrobial prescriptions, which should also be evaluated as a parameter for AMS. Despite these downsides, our data was of help in comprehending the current practice of antimicrobial prescriptions for uncomplicated cystitis, which can be one of the cornerstones of AMS promotion in Japan.

In summary, amid the promotion of AMS to combat AMR, nearly two-thirds of antimicrobials prescribed for uncomplicated cystitis were broad-spectrum agents, primarily fluoroquinolones and third-generation cephalosporins. Male gender, higher age, and visits to the internal medicine department were statistically associated with such prescriptions. Notably, prescriptions of broad-spectrum antimicrobials were not related to the prevention of recurrence. Our present findings would be an indicator for monitoring the antimicrobial prescriptions for patients with uncomplicated cystitis in Japan, which, we expect, can be useful data for health policymakers.

## Methods

### Study design and duration

This was a multicentered, retrospective study of patients who visited the outpatient clinics of six medical institutions in Okayama or Kagawa prefectures in Japan (Okayama University Hospital, Kasaoka City Hospital, Tamano City Hospital, Marugame Medical Center, Tsuyama Chuo Hospital, and Brain Attack Center Ota Medical Hospital) between January 1, 2018, and December 31, 2020.

### Target of the study and data collection

We included outpatients aged ≥ 20 years whose medical records were registered with International Classification of Diseases (ICD-10) codes of N300 that define uncomplicated cystitis (acute cystitis, acute simple cystitis, and acute hemorrhagic cystitis), similar to previous studies^12^. By reviewing the medical records of the patients, we collected data on age, sex, the outpatient department consulted the presence of urinary tract symptoms (frequent urination, dysuria, and pain during urination), urinalysis (leukocyturia, proteinuria, and bacteriuria), bacterial culture, antimicrobial prescriptions, and revisit within one month due to recurrence. We listed the total numbers of isolated pathogens but excluded any of them if three or more organisms were detected in urine. We considered fluoroquinolones, third-generation cephalosporins, and faropenem as broad-spectrum antimicrobials, while we regarded penicillins, first- or second-generation cephalosporins, and sulfamethoxazole-trimethoprim combination as narrow-spectrum antimicrobials. Only medical doctors are authorized to prescribe antimicrobials in Japan but not nurse practitioners or other healthcare professionals. The antimicrobial prescriptions included in this study were not limited to either general practitioners or organ specialists. Patients were categorized into two age groups for the analysis: ≤ 50 years (younger group) or > 50 years (older group). Exclusion criteria were as follows; (1) no clinical symptoms suggestive of cystitis (frequent urination, discomfort during urination, and lower abdominal pain), (2) no urinalysis tested or no abnormalities detected in urinalysis, and (3) no data on consulting department.

### Outcomes

The primary outcome was defined as the prescription rates of broad-spectrum antimicrobials for uncomplicated cystitis in association with factors such as sex, age, and consulting department. The secondary outcome was the association of antimicrobial types (broad-spectrum or narrow-spectrum) with recurrence. The eligible cases were stratified by the age of the patient (< 50 years or ≥ 50 years), sex (female or male), and consulting department (either internal medicine, urology, or others).

### Statistical analysis

Categorical variables were shown in numbers, percentages, and odds ratios (OR) with their 95% confidential intervals (CIs), which were assessed with the Chi-square test or Fisher's exact test as appropriate. Continuous variables were summarized with median and interquartile range (IQR). For multivariate analysis, we applied a logistic regression model. The data were analyzed using EZR software, a graphic user interface for the R 4.0.3 software (The R Foundation for Statistical Computing, Vienna, Austria). All estimates were expressed as point estimates with 95% CI, and all reported *p*-values less than 0.05 were considered statistically significant.

### Ethics approval

The study was approved by the Okayama University’s Graduate School of Medicine, Dentistry and Pharmaceutical Sciences and Okayama University Hospital’s Ethics Committee (No. 1907–036). Informed consent was not necessary because the data were fully anonymized and the waiver of informed consent was approved by the Ethical Committees of Okayama University Hospital (No. 1907–036). Please refer to the following URL for the Ethical Committees of Okayama University Hospital, although it is written in Japanese: http://www.hsc.okayama-u.ac.jp/ethics/files/rk/decision_tree.pdf. The authors assert that all procedures contributing to this work comply with the ethical standards of the relevant national and institutional committees on human experimentation and with the Helsinki Declaration of 1975, as revised in 2008.

## Abbreviations

AMPC: Amoxicillin
AMR: Antimicrobial resistance
AMS: Antimicrobial stewardship
CVA: Clavulanate
ICD: International Classification of Diseases
UTIs: Urinary tract infections
